# Tension-loaded bone marrow stromal cells potentiate the paracrine osteogenic signaling of co-cultured vascular endothelial cells

**DOI:** 10.1242/bio.032482

**Published:** 2018-05-01

**Authors:** Yu Nan Jiang, Jun Zhao, Feng Ting Chu, Yang Yang Jiang, Guo Hua Tang

**Affiliations:** 1 Department of Orthodontics, Ninth People's Hospital, Shanghai Jiao Tong University School of Medicine, Shanghai 200011, People's Republic of China; 2 Oral Bioengineering Lab, Shanghai Key Laboratory of Stomatology & Shanghai Research Institute of Stomatology; National Clinical Research Center of Stomatology, Shanghai 200011, People's Republic of China

**Keywords:** Bone marrow stromal cells, Vascular endothelial cells, Tension, Co-culture, Paracrine signaling

## Abstract

Co-culture of bone marrow stromal cells (BMSCs) and vascular endothelial cells (VECs) is a promising strategy for better osteogenesis and pre-vascularization in bone tissue engineering. Recent reports have shown that mechanical stretching further promotes osteogenesis in BMSC/VEC co-culture systems, but the underlying mechanism of this process remains unclear. In this study, noncontact co-cultures of rat primary BMSCs and VECs were employed to interrogate paracrine cell-to-cell communications in response to tension. Exposure of VECs to 6% tension for 48 h elicited neither ALP activity nor mRNA expression of OCN and OPN in BMSCs incubated in a shared culture medium. Instead, BMSCs subjected to tension induced robust VEGF release, and its conditioned medium enhanced the proliferation and tubular formation of VECs with a concurrent increase in BMP-2 and IGF-1 production. Conditioned medium from activated VECs in turn promoted expression of osteogenic genes in BMSCs, followed by an increase in matrix mineralization. The addition of VEGF-R inhibitor Tivozanib to these systems abrogated the tension-induced paracrine effects on VECs and subsequently impaired BMSC osteogenesis. These results clearly demonstrate that the response of BMSCs to tension potentiates paracrine osteogenic signaling from VECs; this positive feedback loop is initiated by VEGF release.

## Abstract (precis)

**Summary:** This present study demonstrates that the response of BMSCs to tension potentiates paracrine osteogenic signaling from co-cultured VECs; this positive feedback loop is initiated by VEGF release.

## INTRODUCTION

Neovascularization is closely coupled to new bone formation during growth and development as well as wound healing (Saran et al., 2014). Vasculature ingrowth is a prerequisite for endochondral and intramembranous ossification (Filipowska et al., 2017). Vascularization is also crucial to bone tissue regeneration, while an insufficient blood supply always leads to the failure of bone grafts or tissue engineered constructs (Rao and Stegemann, 2013). Various strategies have been employed in bone tissue engineering to overcome this obstacle. There is considerable evidence supporting the co-culture of bone marrow stromal cells (BMSCs) and vascular endothelial cells (VECs) as a promising solution by providing better pre-vascularization and osteogenesis (Grellier et al., 2009a).

In addition to the significance of vascularization, mechanical cues are also important for bone growth and homeostasis (Iolascon et al., 2013). *In vivo*, both BMSCs and VECs are mechanosensitive and constantly exposed to various mechanical stimuli, such as tension, compression, shear stress and hydrostatic pressure (Qin and Hu, 2014). Previous reports indicated that appropriate tension enhanced the osteogenic differentiation of BMSCs, while excessive strain tended to suppress osteogenic differentiation and induce myogenic differentiation (Jang et al., 2011; Steward and Kelly, 2014). On the other hand, cyclic tension also induces morphological alignment in VECs which affects cellular functions such as proliferation, migration, tubular formation and matrix remodeling (Liu et al., 2013). A recent study (Steward et al., 2016) demonstrated the combined effect of 10% cyclic tension and direct co-culture with VECs on BMSC osteogenesis which triggered increased calcium accretion. Our previous work also showed that 6% cyclic tension further promoted osteogenic differentiation in a BMSCs/VECs direct co-culture system (Jiang et al., 2016). Nevertheless, the mechanism by which tension affects BMSCs/VECs behavior and its regulatory mechanism remains to be elucidated.

Many diffusible molecules are involved in the crosstalk between these co-cultured cells. Vascular endothelial growth factor (VEGF) is an important factor for the viability and homeostasis of VECs and BMSCs have been thought to be an abundant source of secreted VEGF proteins (Clarkin et al., 2008). Likewise, VECs have been shown to release growth factors such as endothelin-1 (ET-1), bone morphogenetic protein (BMP-2) and insulin-like growth factor (IGF-1) (Grellier et al., 2009a). ET-1 is a potent vaso-constricting agent involved in BMSC osteogenesis (Qu and von Schroeder, 2006). BMP-2 is closely related to the modulation of osteogenic differentiation (Kaigler et al., 2005), while IGF-1 exerts an anabolic effect on BMSC proliferation and differentiation (Lagumdzija et al., 2004; Reible et al., 2017).

Besides the secretion of diffusible factors, cell-to-cell interactions and gap junction communications are two other mechanisms involved in the crosstalk between bone-forming cells and endothelial cell lineages (Grellier et al., 2009a). Cytoplasmic connections created by gap junctions provide a passage for the direct exchange of ions and small molecules, and these communications have been proven to increase osteogenic biomarkers such as alkaline phosphatase (ALP) in BMSCs (Guillotin et al., 2004). Some reports have demonstrated that crosstalk between BMSCs and VECs under static conditions relied on direct cell-to-cell contacts instead of paracrine communications (Kaigler et al., 2005; Villars et al., 2002). In our recent report we demonstrated that VEGF signaling was involved in tension-induced osteogenesis in a direct BMSCs/VECs co-culture system. In order to determine whether paracrine signaling participates in the regulation of angiogenic/osteogenic activities of BMSC/VEC co-cultures under tension strains, and to further clarify the specific regulatory pathway, we designed noncontact co-culture systems by employing Transwell culture inserts and conditioned medium co-cultures to enable observations of individual cell type behaviors and their complex interactions. Furthermore, a VEGF receptor-specific inhibitor was used to clarify the roles of VEGF in the co-culture system under mechanical stretching.

## RESULTS

### VEC stretching failed to enhance osteogenic potential of indirect BMSC co-cultures

**Fig. 1.**
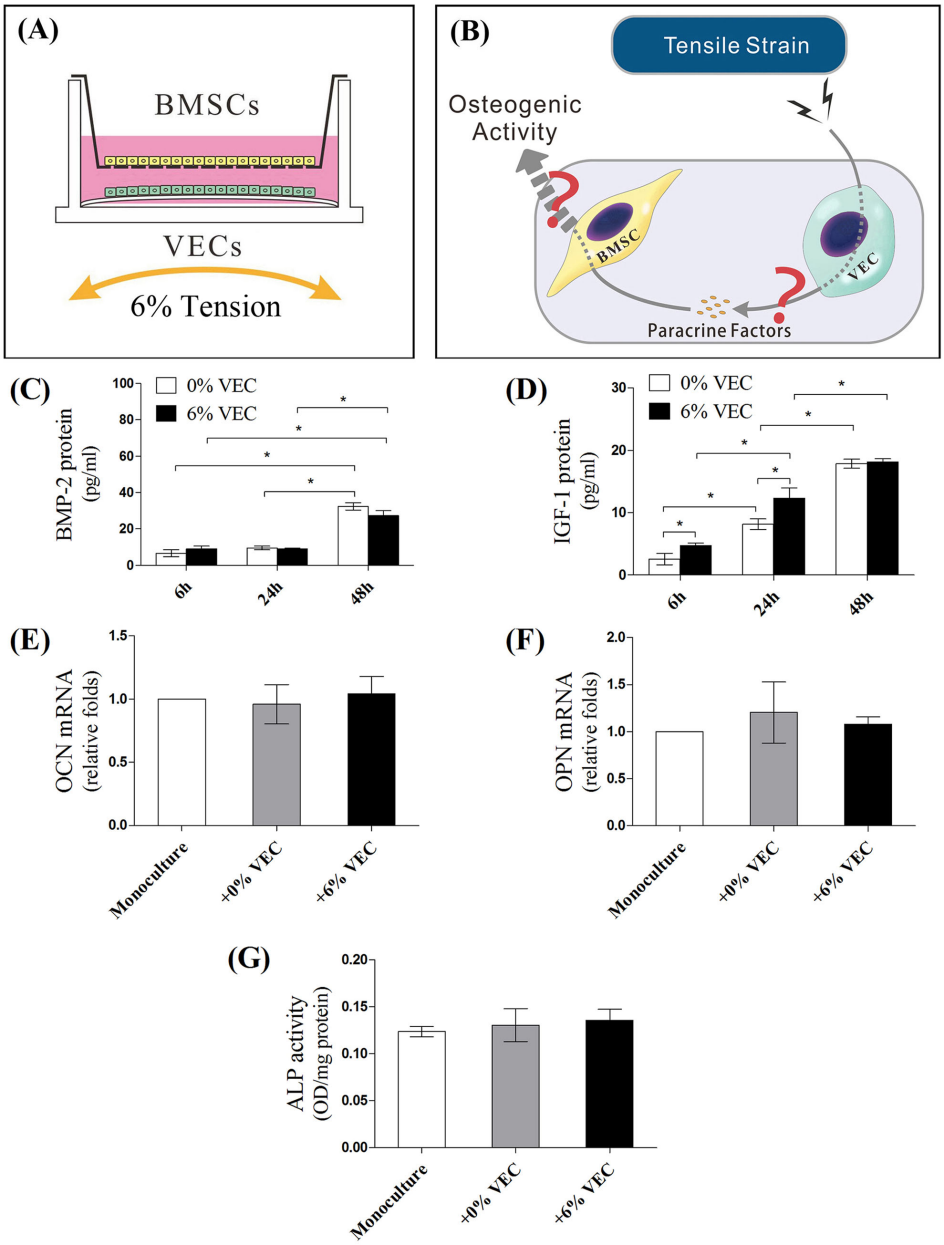
**Transwell indirect co-culture of BMSCs and loaded VECs.** (A) VECs subjected to 6% tension were co-cultured with BMSCs in Transwell insert to analyze (B) the paracrine effects of tension loaded-VECs on the osteogenic activities of BMSCs. Supernatants from mono-cultured VECs were first collected to detect the products of (C) BMP-2 and (D) IGF-1 by ELISA after 6, 24 and 48 h of tension loading. After 48 h of non-contact co-culture with tension-loaded VECs (+6% VEC) or non-loaded VECs (+0% VEC), BMSCs were harvested for mRNA detection of (E) OCN and (F) OPN, and (G) semi-quantitative ALP activity assay (*P<0.05).

In order to test whether VEC stretching could induce the osteogenic differentiation of co-cultured BMSCs through paracrine signaling, VECs were subjected to 6% tension and were co-cultured with BMSCs in a shared culture medium (Fig. 1A,B). Supernatants from VECs monoculture were first collected to detect the products of BMP-2 (Fig. 1C) and IGF-1 (Fig. 1D) under tension loading by enzyme linked immunosorbent assay (ELISA). Although VECs produced more IGF-1 at 6 h and 24 h of stretching, no significant increase in secretions of BMP-2 and IGF-1 was detected at 48 h after loading (Fig. 1C,D). After 48 h of non-contact co-culture with tension-loaded VECs (+6% VEC) or non-loaded VECs (+0% VEC), BMSCs were harvested for measurement of osteogenic markers (Fig. 1E–G). Regardless of loading, VECs had no impact on OCN and OPN mRNA expressions in BMSCs (Fig. 1E,F). Similarly, co-cultured BMSCs exhibited no significant difference in ALP activity compared to monoculture controls (Fig. 1G). These results suggested that VECs, in static or loading conditions, could not secrete diffusible factors sufficient to promote osteogenic activity in BMSCs.

### Tension elevated VEGF secretion in BMSCs and promoted osteogenesis via VEC-mediated paracrine signaling

**Fig. 2.**
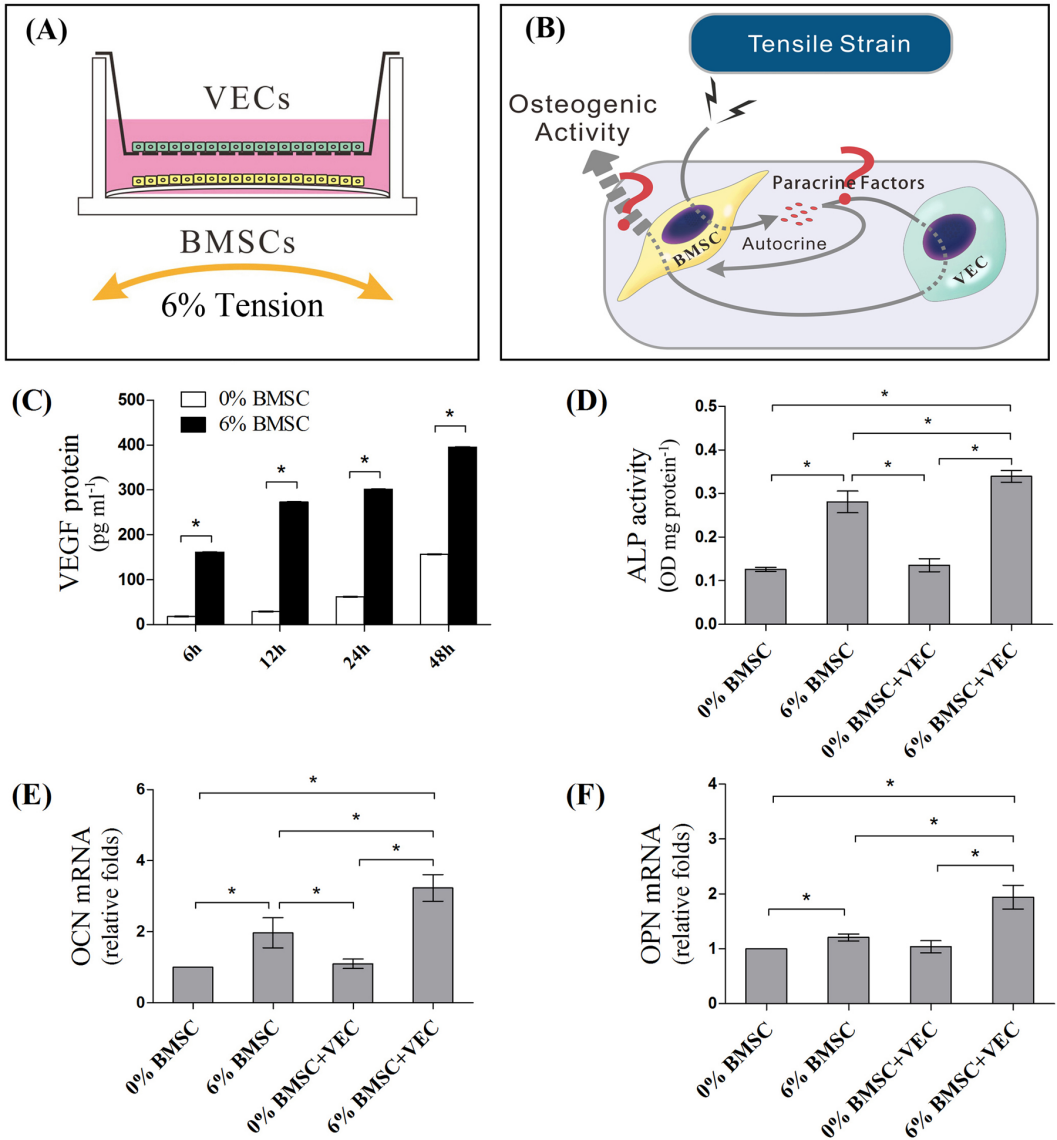
**Transwell indirect co-culture of VECs and loaded BMSCs.** (A) BMSCs subjected to 6% tension were co-cultured with VECs in Transwell insert to analyze (B) the paracrine effects of tension loading on the osteogenic activities of BMSCs mediated by VECs. Supernatants from monocultured BMSCs were first collected to detect the products of (C) VEGF by ELISA after 6, 12, 24 and 48 h of tension loading. After 48 h of non-contact co-culture with VECs, the loaded BMSCs (6% BMSCs+VEC) or non-loaded BMSCs (0% BMSCs+VEC) were harvested for (D) semi-quantitative ALP activity assay and mRNA detection of (E) OCN and (F) OPN. The mono-cultures of loaded and non-loaded BMSCs served as controls (0% BMSCs and 6% BMSC) (*P<0.05).

Loading of VECs did not initiate an osteogenic effect in BMSCs via the paracrine pathway, therefore the synergic effects of tension and VECs on BMSCs as previously reported might be triggered by the BMSC response to mechanical force. Thus, BMSCs subjected to 6% tension were co-cultured with VECs using a Transwell insert (Fig. 2A,B). ELISA assay results showed that tension induced a significant production of VEGF in the supernatant of BMSCs monoculture as early as 6 h after loading. There was a 2.5-fold increase in VEGF levels detected at 48 h after loading in the tension culture when compared to static controls (Fig. 2C). After 48 h loading, BMSC monoculture also showed a significant increase in ALP activity, OCN and OPN mRNA expression compared to the static cells (Fig. 2D–F, 0% BMSC vs 6% BMSC). When the loading BMSCs were co-cultured with VECs, more significant increases in the expressions of osteogenic markers were observed (Fig. 2D–F, 6% BMSC+VEC vs 0% BMSC+VEC). In contrast, co-culture with VECs and non-loading BMSCs failed to enhance the osteogenic activities when compared to non-loading monoculture BMSCs (Fig. 2D–F, 0% BMSC+VEC vs 0% BMSC). These results indicated that tension promoted osteogenic differentiation of BMSCs possibly by an autocrine signaling and/or a VEC-mediated paracrine pathway; VEGF signaling may also be involved in this process.

### BMSCs transduced tension and activated VECs via VEGF signaling

**Fig. 3.**
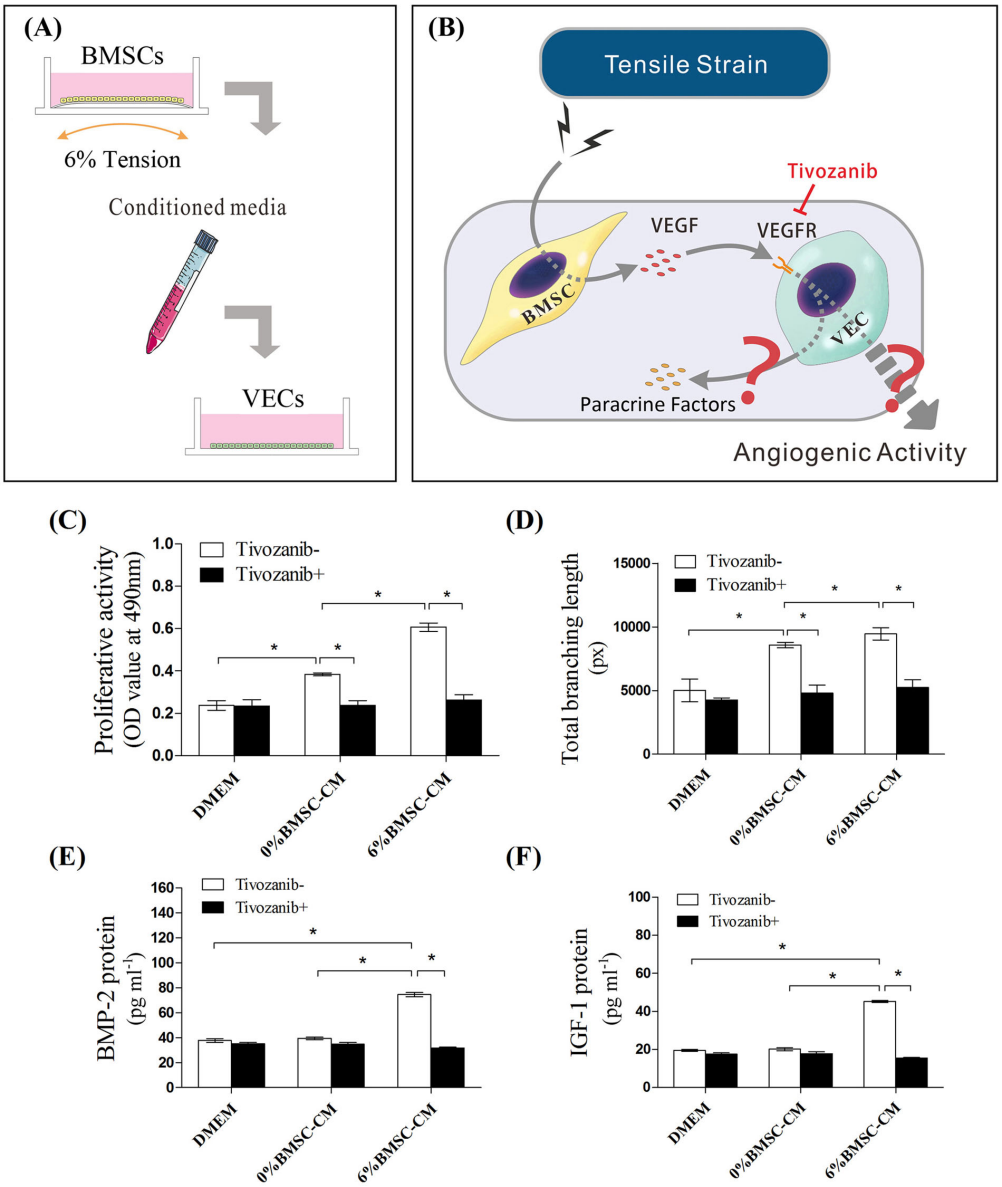
**Conditioned media co-culture of VECs.** (A) Conditioned media from loaded BMSCs was harvested for the culture of VECs to analyze the paracrine effects of (B) tension-induced VEGF signaling on the activities of VECs using VEGF-R inhibitor Tivozanib. VECs suspended in conditioned media from loaded (6% BMSC-CM) or non-loaded BMSCs (0% BMSC-CM) were either (C) cultured for 48 h for MTT assay, or (D) plated on Matrigel Matrix for 12 h, with total branching length measured for the evaluation of angiogenic activity. Cultures in DMEM served as control. After 48 h of culture, the conditioned medium was replaced by fresh DMEM for another 48 h incubation, then (E) BMP-2 and (F) IGF-1protein levels in the supernatants were detected by ELISA (*P<0.05).

In order to clarify the VEC response to loaded BMSC-induced VEGF signaling, VECs were incubated in the conditioned medium from loaded BMSCs (6% BMSC-CM) for 48 h. Afterwards, the VECs were analyzed with or without treatment of VEGF-receptor (VEGF-R) inhibitor Tivozanib (Fig. 3A,B). The Matrigel angiogenesis assay was conducted at 12 h (Fig. 3D), while the proliferative and paracrine activities of VECs were examined at 48 h (Fig. 3C,E,F). MTT assay showed that incubation in conditioned medium from BMSCs (0% BMSC-CM) increased the proliferation of VECs compared to the DMEM control group. A further increase in proliferative activities was observed in the 6% BMSC-CM-treated group (Fig. 3C). Similarly, 6% BMSC-CM promoted angiogenic activity in VECs, with a significant increase in branching length and tubular formation (Fig. 3D, Fig. S1). Meanwhile, ELISA assay results showed that 6% BMSC-CM-treated VECs expressed significantly more BMP-2 and IGF-1 proteins. Treatment with conditioned medium from non-loaded BMSCs, however, had no difference on BMP or IGF secretions compared to the DMEM groups (Fig. 3E,F).

When treated with VEGF-R inhibitor, the proliferative activity of VECs was decreased by 38% and 57% in 0% BMSC-CM group and 6% BMSC-CM group, respectively (Fig. 3C). Similarly, Tivozanib blocked any angiogenic effects in both 0% BMSC-CM and 6% BMSC-CM groups, which included discontinuous tubular structures in the Matrigel angiogenesis assay (Fig. S1) and a decrease in total branching length compared to baseline in the DMEM groups (Fig. 3D). The paracrine activities of VECs in the 6% BMSC-CM group were also inhibited by Tivozanib, with a decreased production of BMP-2 and IGF-1 (Fig. 3E,F).

### Tension enhanced osteogenic effects of BMSCs via autocrine and VEC-mediated paracrine pathways

**Fig. 4.**
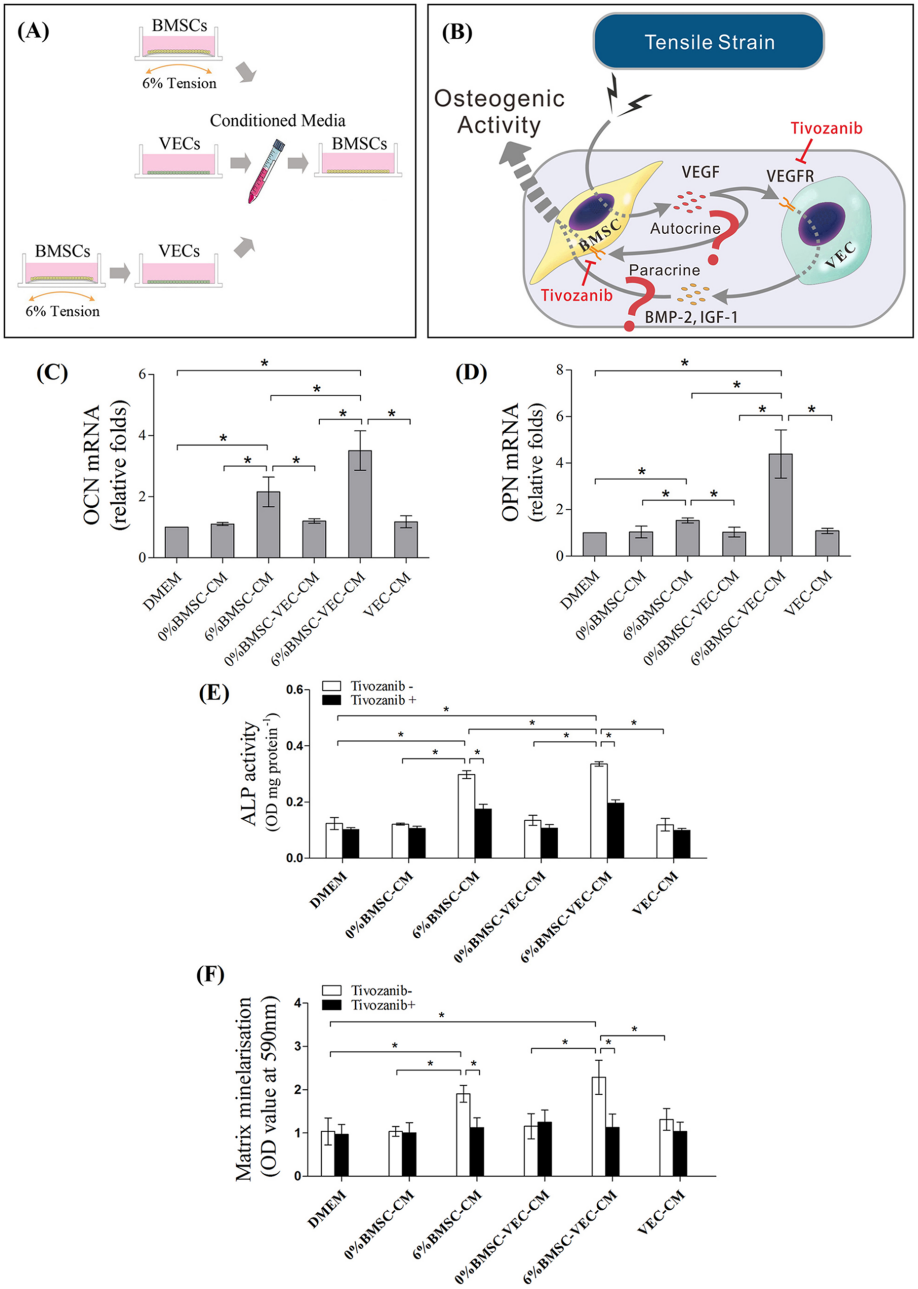
**Conditioned media co-culture experiments of BMSCs.** (A) Conditioned media from VECs (VEC-CM), loaded BMSCs (6% BMSC-CM), and pretreated VECs with loaded BMSC-CM (6% BMSC-VEC-CM) were harvested for the culture of BMSCs to analyze (B) the paracrine and autocrine effects of tension-induced VEGF signaling on the osteogenic activities of BMSCs by using the VEGF-R inhibitor Tivozanib. BMSCs were incubated in the above conditioned media for 48 h to quantify mRNA expression of (C) OCN and (D) OPN, 7 days for (E) ALP activity, and (F) 14 days to assess matrix mineralization. DMEM or conditioned media from non-loaded counterparts (0% BMSC-CM and 0% BMSC-VEC-CM) served as controls (*P<0.05).

Although the paracrine effect of VEGF on VECs has been proven, whether activated VECs in turn promote BMSC osteogenesis remained to be verified. To this end, the culture medium from VECs (VEC-CM), loaded BMSCs (6% BMSC-CM), and pretreated VECs with loaded BMSCs (6% BMSC-VEC-CM) were used to culture BMSCs for 48 h and afterwards the osteogenic activities of BMSCs were examined (Fig. 4A,B). VEC-CM or 0% BMSC-CM had no impact on BMSC osteogenesis, while 6% BMSC-CM led to a 2.7-fold increase in ALP activity, higher OCN and OPN mRNA expression at 48 h, and more matrix mineralization on day 14 (Fig. 4C–F, *P*<0.05). These results indicated that tension promoted BMSC osteogenic differentiation, at least partially, via an autocrine pathway.

When VECs were stimulated by BMSC-CM, the conditioned medium significantly increased osteogenic effects in BMSCs. The 6% BMSC-VEC-CM group exhibited the highest ALP activity and increased matrix mineralization, as well as having the most abundant OCN and OPN mRNA expressions (Fig. 4C–F, *P*<0.05). Tivozanib treatment of VECs blocked this osteogenic effect. The ALP activity of the 0% BMSC-VEC-CM group showed a 21% decrease compared to untreated control, while 6% BMSC-VEC-CM group exhibited a 42% decrease (Fig. 4E, *P*<0.05).

## DISCUSSION

To achieve better pre-vascularization and osteogenesis, the BMSCs/VECs co-culture system has been employed in bone tissue engineering (Kaigler et al., 2005; Xue et al., 2009). Meanwhile, tension was also found to play an important role in regulating the osteogenic differentiation of BMSCs (Steward and Kelly, 2014). The synergic effects of mechanical stimuli and BMSCs/VECs co-culture on osteogenic and angiogenic activities has been recently reported. One study (Jimenez-Vergara et al., 2013) co-cultured BMSCs and VECs in a tubular scaffold which was then subjected to tension generated by pulsatile flow. They found the combined effect of VEC-presence and tension to further enhance the rate and degree of BMSC osteogenesis. Another recent study (Steward et al., 2016) found that directly co-cultured BMSCs/VECs showed a significant increase in calcium accretion and mineral deposition under tension. Similarly, in our previous research we observed a synergic effect of 6% tension and directly co-cultured VECs on BMSC osteogenesis, which exhibited enhanced ALP activity and an increase of runt-related transcription factor 2 (Runx-2) mRNA (Jiang et al., 2016). However, the roles BMSCs and VECs played in the loaded co-culture systems have yet to be clarified. In the present study, we demonstrated that the synergic effects of tension and VEC co-culture on the osteogenic potential of BMSCs was initiated by the response of BMSCs towards the mechanical strains at least in part through autocrine and VEC-mediated paracrine signaling.

Both BMSCs and VECs are mechanosensitive cells. Mechanical stimuli not only induces morphological alignment in VECs, but also affects VEC survival and intracellular signaling pathways, producing a variety of growth factors and cytokines, such as palate-derived growth factor, basic fibroblast growth factor and transforming growth factor-β (Liu et al., 2013). It is reasonable to assume that VECs activated by tension could promote BMSC osteogenesis via paracrine pathways. VEC monocultures were first subjected to 6% tension and the production of BMP-2 and IGF-1 in the culture medium were detected. ELISA assay showed an increase in IGF-1 secretion upon 6 h and 24 h of stretch loading. However, the increase disappeared at 48 h of loading (Fig. 1D). No significant change of BMP-2 expression was detected (Fig. 1C). This transient elevation of IGF-1 failed to promote the osteogenic differentiation of BMSCs in the noncontact co-culture system (Fig. 1A,E–G). It is possible that the mechanical loading regimen of 6% tension used was not sufficient to elevate significant amounts of VEC diffusible factors. According to previous reports, however, lower tension (5%) increased, but higher tension (20%) decreased, VECs survival and angiogenesis (Kou et al., 2009). In our previous studies, 6% tension but not 3% or 9% was proved to be optimal in inducing BMSC/VEC osteogenesis (Jiang et al., 2016). These results suggested that VECs, under static or loading conditions, could not secrete enough diffusible factors sufficient to prompt osteogenic activity in co-cultured BMSCs.

Since VECs were not observed to be a major mechano-transductor in our co-culture model, it could be the responses of BMSCs towards tension initiated a series of osteogenic/angiogenic effects. When BMSCs were subjected to 6% tension, the mechanical loading itself (6% BMSC group) effectively enhanced BMSC osteogenesis (Fig. 2D–F). When VECs were involved in the system via shared culture medium, a synergic effect upon BMSC osteogenic activities was observed by mechanical loading (6% BMSC+VEC group), as indicated by further increased ALP activity and osteogenic gene expressions (Fig. 2D–F). These results suggested that BMSCs were activated by tension, and tension-induced growth factors or cytokines from BMSCs might trigger crosstalk between BMSCs and VECs via paracrine pathways.

We then focused on the production and function of VEGF, because our previous reports and others showed that VEGF expression in BMSC/VEC direct co-culture was elevated by tension strain and that BMSCs accounted for VEGF production (Grellier et al., 2009b; Jiang et al., 2016; Zhu et al., 2015). Upon stretch loading, significant increases in VEGF secretion by BMSCs were consistently detected from 6 h to 48 h (Fig. 2C). Conditioned medium from static BMSCs (0% BMSC-CM) promoted the proliferation and tubular formation of VECs (Fig. 3C,D). These angiogenic effects were more significant when VECs were incubated in loaded BMSCs medium (6% BMSC-CM, Fig. 3C,D). When VECs were treated with VEGF-R inhibitor, the paracrine effects of BMSC-CM were abrogated under both static and loading conditions (Fig. 3C,D). These results implied that the activation of VECs by BMSCs co-culture, whether loaded or not, was mediated by VEGF signaling.

BMSC osteogenesis was enhanced by supernatants from VECs treated by 6% BMSC-CM (Fig. 4C–F). VECs are responsible for the production of various osteogenic growth factors which compose a complicated paracrine network (Grellier et al., 2009a). We chose to focus on BMP-2 and IGF-I because they are potent to induce osteogenesis in BMSCs. The effect of VECs on BMSCs ALP activity was mimicked by adding recombinant BMP-2 proteins (Kaigler et al., 2005). On the other hand, when the expression of BMP-2 was inhibited by small interfering RNA in VECs and then co-cultured with BMSCs, a large decrease was found in the osteogenic differentiation (Kaigler et al., 2005). Similarly, IGF-1 treatment enhances the osteogenic differentiation of BMSCs (Reible et al., 2017). Thus, such osteogenic effects of 6% BMSC-VEC-CM in our current study were based at least in part on the elevation of VEC-source growth factors such as BMP-2 and IGF-1 (Fig. 3E,F). Addition of Tivozanib abrogated the paracrine functions of VECs on the productions of BMP-2 and IGF-1, and subsequently impaired BMSC osteogenic activities (Figs 3E,F, 4E). These data suggested that the osteogenic effects of VECs on BMSCs also depended on VEGF signaling. Of note, the osteogenic effects of conditioned medium from loaded BMSCs (6% BMSC-CM) were also abolished by Tivozanib treatment (Fig. 4E). This suggested that autocrine signaling existed when BMSCs were subjected to tension, which was also mediated by VEGF.

In addition to the paracrine/autocrine signaling of diffusible molecules, communication between osteogenic and endothelial cell lineages also depend on direct cell-to-cell interactions, such as adherens, tight junctions and gap junction communications (Grellier et al., 2009a). The osteogenic effect of BMSCs/VECs co-culture with direct contact has been demonstrated by various reports (Grellier et al., 2009b; Guillotin et al., 2004). It was important to note in our present study that the baseline of VEGF production by non-loaded BMSCs activated VEC proliferation and angiogenic activities (Fig. 3C,D, 0% BMSC-CM versus DMEM). This amount of VEGF however, was not enough to elicit BMP and IGF production (Fig. 3E,F, 0% BMSC-CM versus DMEM). As a result, the osteogenic activity of BMSCs were not increased by indirectly co-cultured VECs (culture inserts) or conditioned medium under static conditions (Figs 1E–G, 4C–F). These results indicated that VECs could not promote BMSC osteogenesis via paracrine pathways, which was in accordance with previous reports (Kaigler et al., 2005). According to research by Villars et al. (2002), communications between BMSCs and VECs under static conditions relied on direct cell-to-cell contact (e.g. gap junctions), which might explain such a phenomenon. The response of BMSCs under tension triggered crosstalk between BMSCs and VECs via paracrine signaling, and these paracrine factors completed a positive feedback loop between both cell types. Such a network of paracrine signaling pathways accounted for the synergic osteogenic/angiogenic effects under mechanical stretching and may be a regulatory mechanism independent from that of direct contact co-culture under static conditions. Nevertheless, whether and how the direct cell-to-cell interactions between BMSCs and VECs respond to mechanical stimuli awaits further studies.

In summary, the present study revealed paracrine crosstalk between BMSCs and VECs in response to mechanical loading (Fig. S2). Stretching the VECs failed to secrete enough diffusible factors sufficient to trigger the osteogenic activities of BMSCs. Stretching the BMSCs, on the other hand, induced VEGF secretion. VEGF enhanced angiogenic activities in VECs and induced the release of osteogenic factors, like BMP-2 and IGF-1, which in turn promoted BMSC osteogenesis. Although direct cell interactions are indispensable in cell-to-cell communications, our results reiterated VEGF signaling as creating a positive feedback loop between BMSCs and VECs. Understanding of these intrinsic regulatory mechanisms will provide new insights into adaptive bone remodeling as well as bone tissue engineering. Data from our present works suggest that the manipulation of VEGF signaling, either by mechanical input upon BMSCs or by cooperating VEGF proteins to mimic the effect of mechanical cues, could be a promising strategy for better osteogenesis and angiogenesis during bone regeneration. Further research concerning mechanical loading regimes and *in vivo* verification are required for potential clinical applications of this co-culture system.

## MATERIALS AND METHODS

### Cell isolation and culture

Rat BMSCs were isolated and expanded using previously reported methods (Maniatopoulos et al., 1988). Animals were purchased from the animal experimental center of the Shanghai Ninth People's Hospital, and experiments were approved by Shanghai Jiao Tong University [Approval No. HKDL(2017)86]. Briefly, femora were obtained from 4-week-old male Sprague-Dawley rats, and bone marrow was then flushed out using complete Dulbecco's modified Eagle medium (DMEM, HyClone, Logan, USA), supplemented with 10% fetal bovine serum (FBS, Gibco) and 1% penicillin/streptomycin (HyClone). Cells were plated on a culture dish, changing DMEM twice a week. After one week of incubation, BMSCs were regularly subcultured and cells were used for experiments between their 2nd–4th passages.

For isolation and culture of rat VECs, an explant culture method was employed (Dariima et al., 2013). The descending aortas of rats were cut into 1–2 mm pieces, and then placed in a 60 mm culture dish with the endothelial surface face down. The explant tissues were incubated in endothelial cell growth medium-2 (EGM-2, Lonza, Basel, Switzerland) for 9 days, and then removed. VECs were regularly subcultured and cells were used for experiments between their 2nd–4th passages.

### Application of tension

Cells were seeded on six-well Bioflex culture plates (coated with type I collagen, Flexcell, Burlington, USA) at 5×10^4^ cells ml^−1^ and then incubated for 72 h in DMEM until 90% cell confluence. Subsequently, culture plates were subjected to equibiaxial cyclic tension (0.5 Hz, semi-sinusoidal wave form) at 6% elongation by Flexcell 5000 Tension System (Flexcell). This loading regimen was shown to be most effective in inducing BMSC osteogenic differentiation in our previous reports (Jiang et al., 2016). At 48 h after loading, samples were harvested for an assay of ALP activity, ELISA and quantitative reverse transcription polymerase chain reaction (qRT-PCR).

### BMSC/VEC indirect co-culture

Transwell culture inserts in six-well plates (0.4 μm-pore-size polyester membrane, Corning, New York, USA) were employed to establish an indirect co-culture system. Briefly, BMSCs and VECs were trypsinized and re-suspended in complete DMEM at a density of 5×10^4^ cells ml^−1^. Either BMSCs or VECs were seeded in Transwell inserts at 5×10^4^ cells ml^−1^, while cells of the other type were plated on Bioflex plates (Figs 1A, 2A). Cells were incubated independently for 72 h until confluent and then indirectly co-cultured via shared culture media for an additional 48 h with or without tension loading.

Conditioned media (CM) were harvested for indirect co-culture in compensation of Transwell co-culture model, so as to further elucidate the one-way paracrine pathway regulating angiogenic/osteogenic activities. Briefly, upon cell confluence culture media of BMSCs were replaced with fresh DMEM, and cells were then subjected to 6% tension for 48 h. Conditioned media from loaded (6% BMSC-CM) or non-loaded (0% BMSC-CM) BMSCs were harvested for the culture of VECs, with conventional complete DMEM served as a control (Fig. 3A).

After VECs were stimulated by BMSC-CM for 48 h, the culture media were replaced with fresh DMEM for another 48 h incubation, and then conditioned media from pretreated VECs (6% BMSC-VEC-CM) or non-treated VECs (VEC-CM) were harvested for BMSC culture to analyze the paracrine pathway. Meanwhile, 6% BMSC-CM were also used for BMSCs culture to analyze the autocrine pathway. DMEM or conditioned media from non-loaded counterparts (0% BMSC-CM and 0% BMSC-VEC-CM) served as controls.

### qRT-PCR

The total RNA was extracted from cell samples by acid-guanidine-phenol-chloroform extraction using RNAiso Plus reagent (Takara, Kusatsu, Japan) and was then quantified with a Thermo Fisher Scientific NanoDrop™ 1000 ultraviolet-visible spectrophotometer (Thermo Fisher Scientific). A total of 1 μg RNA was used as template for single-strand cDNA synthesis using a PrimeScript RT reagent Kit (Takara). After reverse transcription, real-time PCR was performed in a 20 μl standard reaction system (SYBR PrimeScript RT-PCR Kit, Takara) by Bio-Rad IQ5 Real-Time PCR System (Bio-Rad). The cycle threshold (CT) values were used to calculate the relative fold-change based on the value of each control sample (2^−ΔΔCT^ method). β-actin served as an internal normalized reference. Melting curve analysis was performed to evaluate specificity of primers. Primers used are listed below:

OCN-F, 5′-GCCCTGACTGCATTCTGCCTCT-3′

OCN-R, 5′-TCACCACCTTACTGCCCTCCTG-3′

OPN-F, 5′-CCTTCACTGCCAGCACACAA-3′

OPN-R, 5′-CTGTGGCATCGGGATACTGTT-3′

β-actin-F, 5′-GTAAAGACCTCTATGCCAACA-3′;

β-actin-R, 5′-GGACTCATCGTACTCCTGCT-3′.

### ELISA

Supernatants collected from BMSC and VEC culture were subjected to ELISA assay for the measurements of paracrine factors. The VEGF, BMP-2 and IGF-1 secretions of each group were quantified by sandwich technique using the rat VEGF ELISA kit, rat BMP-2 ELISA kit and rat IGF ELISA kit (Biotechwell, Shanghai, China) according to the manufacturer's instructions. The absorbance was measured at 450 nm (DENLEY DRAGON Wellscan MK3, Thermo Fisher Scientific), and protein concentrations were calculated according to a standard curve.

### Assessment of ALP activity

For the semi-quantitative assessment of ALP activity, BMSCs were washed twice with PBS and lysed in 450 μl of ALP lysis buffer (double distilled water containing 0.2% Triton-100, 9.7% ethanolamine, 0.2 mg ml^−1^ sodium azide, 0.1 mg ml^−1^ MgCl_2_, 1 mM HCl). Total protein was then quantified using a BCA Kit (Thermo Fisher Scientific). Subsequently, 100 μl 1 mg ml^−1^ p-nitrophenylphosphate (dNPP, Sigma-Aldrich) was added to 100 μl of cell lysate as a substrate and then incubated for 30 min at 37°C. Absorbance was measured at 405 nm (BioTek ELx800, Winooski, USA), and ALP activity was expressed as an OD value per mg protein.

### Matrix mineralization

To evaluate matrix mineralization, Alizarin Red staining was performed using a previously reported method (Heo et al., 2010). BMSCs were fixed in 4% paraformaldehyde for 30 min and then washed twice by PBS. Subsequently, cells were stained with 1 ml 0.1% Alizarin Red S working solution (Cyagen Biosciences, Santa Clara, USA) for 5 min. Cells were rinsed with PBS again and then observed under a light microscope. For semi-quantitative assay, 500 μl 100 mg ml^−1^ cetylpyridinium chloride (Sigma-Aldrich) were added to each well. After incubation for 1 h, the supernatants were transferred to a 96-well plate, and the absorbance was measured at 590 nm (BioTek ELx800).

### MTT assay

For assessment of cell proliferative activity, VECs were subjected to MTT assay using a MTT Cell Proliferative and Cytotoxicity Assay Kit (Beyotime, Shanghai, China). In brief, after 48 h of incubation in BMSC-CM, a total of 10 μl of 5 mg ml^−1^ MTT solution was added to each well of a 96-well plate and cells were incubated for another 4 h for formazan formation. Subsequently, formazan was dissolved in 100 μl dimethyl sulphoxide (DMSO) and the absorbance was read at 490 nm (BioTek ELx800).

### Matrigel angiogenesis assay

The Matrigel angiogenesis assay was performed as previously described (Evans, 2015). Briefly, 100 μl of Matrigel (BD Biosciences) was added to each well of an ice cold 96-well plate and solidified at 37°C for 30 min. Then VECs cultured in complete DMEM culture media or conditioned media were seeded to the plates at 1×10^4^ cells per well. Cells were incubated for 12 h, and images were obtained using an inverted phase contrast microscopy (Olympus IX71 Microscope and DP71 Camera, Tokyo, Japan). ImageJ 1.50 software (Wayne Rasband International Institutes, Bethesda, USA) was used to analyze the total branching length of tubular structures.

### VEGF-R inhibitor treatment

For analysis of VEGF paracrine pathway, VECs in DMEM or in conditioned media from BMSCs (0% BMSC-CM or 6% BMSC-CM) were treated with 1 μM VEGF-R inhibitor Tivozanib (Selleck, Houston, USA). Cell counterparts with no inhibitor treatment served as the control. The Matrigel angiogenesis assay was conducted at 12 h after inhibitor treatment, while proliferative and paracrine activities of VECs were assessed at 48 h. After Tivozanib treatment for 48 h, culture media of VECs were replaced with fresh DMEM for another 48 h incubation, and conditioned media (VEC-CM, 0% BMSC-VEC-CM and 6% BMSC-VEC-CM) were harvested for BMSC culture. Meanwhile, for analysis of the VEGF autocrine pathway, BMSCs in DMEM or in conditioned media from BMSCs (0% BMSC-CM or 6% BMSC-CM) were treated with 1 μM Tivozanib. Counterparts with no inhibitor treatment served as the control. ALP activity in BMSCs were examined at 48 h after Tivozanib treatment.

### Statistical analysis

All experiments were repeated in triplicate, and the data are presented as the mean±standard deviation. Statistical analysis was performed using Student's *t*-test for the two specific groups of interest with SPSS 19.0 (IBM SPSS Statistics, IBM). A confidence level of *P*<0.05 was determined to be statistically significant.

## Supplementary Material

Supplementary information
