# Supplementary material for: Tension-loaded bone marrow stromal cells potentiate the paracrine osteogenic signaling of co-cultured vascular endothelial cells
Source: Biol Open. 2018 May 1;7(6):bio032482. doi: 10.1242/bio.032482 (PMC6031349; doi:10.1242/bio.032482)
Supplement: Supplementary information [file biolopen-7-032482-s1.pdf]

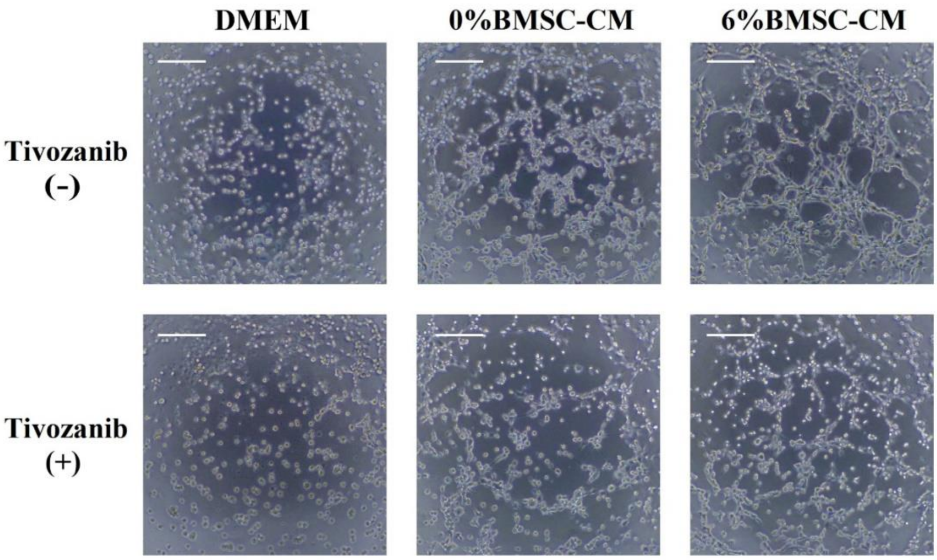

Figure S1. Matrigel angiogenesis assay for VECs cultured in conditioned media. VECs in conditioned media from loaded BMSCs (6% BMSC-CM) or non-loaded BMSCs (0% BMSC-CM) were plated on Matrigel matrix and cultured for 12 h for angiogenic activity assessment. VECs in DMEM served as control. Tivozanib was added to block VEGF signaling. Images were captured at a magnification of 400X (Leica DM IRB microscope, Germany). Quantitative analysis of tubular structures was performed and results are shown in Fig. 3D (Scale bars: 20 μm).

Biology Open • Supplementary information

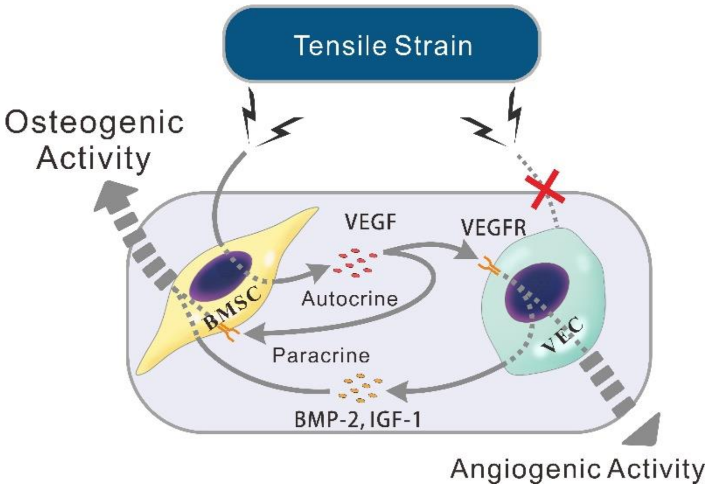

Figure S2. Schematic representation of the paracrine and autocrine signaling pathways of BMSCs and VECs in response to tension based on our current study results. Upon tensile strain, VECs failed to secrete enough diffusible factors sufficient to trigger the osteogenic activities of BMSCs. BMSCs, on the other hand, induced VEGF secretion under mechanical stretching. The upregulation of VEGF promoted BMSC osteogenesis directly or through activating VECs to release osteogenic factors, like BMP-2 and IGF-1.
